# Construction Notes

**Published:** 1907-09-21

**Authors:** 


### CONSTRUCTION NOTES.

It has been decided thoroughly to overhaul, and, if necessary, to reconstruct, the entire drainage system of the Norfolk and Norwich Hospital. The cost of the renovations is estimated at £2,000.

A cottage hospital, to be called "The John Martin Hospital," recently erected at Uig, on the Congested Districts Board's estate, by the trustees of the late Mr. John Martin, Treaslane, formerly of Ceylon, has just been opened. Mr. Martin bequeathed the residue of his estate, now amounting to £10,000, for founding and endowing the hospital. The buildings and furnishings have cost £1,200. The hospital is erected on a rising ground overlooking Uig Bay, has a southern exposure, and consists of two wings, providing two wards with three beds in each, with suitable lavatory and other accommodation.

The Preston, Fulwood, and Longridge Joint Hospital, the scheme for which was first discussed over ten years ago, has at last been opened. It cost over £14,000, and is situated at the junction of Long Sand Lane, Fulwood, and the highway leading to Haighton. The buildings now erected are the administrative block, the scarlet fever block, and the isolation pavilion, and there are left spaces for the building of four additional scarlet fever blocks and typhoid and diphtheria blocks, but this portion of the scheme will only be proceeded with as their erection is deemed necessary. The present scarlet fever block contains male and female wards for eight beds each, and there are also two private wards. In the isolation pavilion there are likewise two wards for two beds each. The architects were Messrs. Seward and Rawcliffe, and the contractor the late Mr. T. Cottam.

Handel Cossham Memorial Hospital at Kingswood recently opened, stands on the highest point in the eastern suburb of Bristol. It is early Georgian in style of architecture, rectangular in shape, and is built of red pennant, with Bath stone dressings; the roof of the main building is covered with Cumberland slates, and the wood and iron facings are painted green. The main building is of three stories and basement, the ground and first floor being used for hospital purposes. Here is the administrative department; the wards are at the western end; and a moderate-sized isolation section is at the eastern end. The entrance on the south side is of attractive design, and above the architrave is the inscription, "Handel Cossham Memorial Hospital, 1905," the arms of the donor surmounting it; while on either side, over the bow windows of the board and matron's rooms, are his crest and mottoes. The internal arrangements and the decorations and fittings are of the latest design. On the ground floor there is a receiving-room for patients, adjoining it is the matron's room, and at the east end wards for cases needing to be isolated. To the left of the entrance are the board-room, surgeons' sitting-room, surgery, and laboratory; and at the west end are surgical wards and patients' department. On the same floor are a fine operating-theatre, adjoining which is an anæsthetic and sterilising room. On the upper floor are matron's and nurses' bedrooms and sitting-rooms; at the west end are the two splendid medical wards, fifty feet by twenty-six feet each, planned to receive ten beds and two cots—twenty-two in all. Adjoining are bath-rooms, lavatories, etc. At the east end are two other isolation wards, perfectly arranged and fitted, so far as modern invention goes. There are also provided spacious day-rooms for patients, kitchen, larder, and all other adjuncts necessary for a hospital of the highest class. The warming and ventilation have received particular attention. Blundel Spence's petrifying liquid has been used to cover the walls and ceilings of the wards, corridors, and other parts of the building. When dry it is white, hard, and shines like glass. All the angles of the apartments and corridors are rounded, that no germs may find a lodgment. Fire hydrants are placed at various points. In the wards are Musgrave's open-fire stoves, in addition to water-pipes for heating purposes. The floors of the wards are of teak blocks laid on concrete. In the operating-theatre and rooms adjoining the floors, walls, and ceilings are of Brit-opal.

